# Vesicle dynamics in synapsin-induced condensates by passive X-ray microrheology

**DOI:** 10.1016/j.bpj.2026.03.006

**Published:** 2026-03-06

**Authors:** Titus Czajka, Andras Major, Hendrik Bruns, Marco Cammarata, Christian Hoffmann, Dragomir Milovanovic, Tim Salditt

**Affiliations:** 1 Institute for X-ray Physics, Friedrich-Hund-Platz 1, 37077 Göttingen, Lower Saxony, Germany; 2 ESRF - European Synchrotron Radiation Facility, 71 Avenue des Martyrs, 38000 Grenoble, Rhone-Alpes, France; 3 DZNE - German Center for Neurodegenerative Diseases, Virchowweg 6, Berlin 10117, Germany; 4 Institute of Biochemistry, Charité-Universitätsmedizin Berlin, Corporate Member of Freie Universität Berlin, Humboldt-Universität Berlin, and Berlin Institute of Health, Berlin, Germany

## Abstract

The collective dynamics of subcellular biological processes is often difficult to assess experimentally due to the challenges associated with spatial and temporal resolution, labeling, or multiple scattering. X-ray photon correlation spectroscopy is, in principle, well suited to probe collective dynamics by quantifying dispersion relations in complex fluids in general and biomolecular systems in particular. However, the low scattering signal and the sensitivity to radiation damage set stringent limits to many applications. Probing the dynamics of vesicles in protein-induced condensates is a case in point. Here, we use lipid vesicles with a hard silica core, called colloid-supported lipid bilayers, as labeled vesicles for enhanced X-ray contrast. We then probe structure and dynamics in solutions of vesicles and synapsin, a protein known for its property of inducing liquid-liquid phase separation and forming condensates that recruit vesicles, organizing them into clusters in presynaptic nerve terminals. The dynamics in these systems is found to exhibit evidence for both liquid-like and network-like phases. Our results reveal distinct effective-diffusion constants at varying protein concentrations. At the same time the stretched exponential decay of the correlation functions provides clear evidence for nondiffusive behavior within the condensates.

## Significance

We introduce colloid-supported lipid bilayers (CSLBs) as high-contrast probes for X-ray photon correlation spectroscopy (XPCS) and use them to probe the collective dynamics in synapsin-vesicle condensates, which can be regarded as a model system for synaptic vesicle pools. The XPCS analysis reveals distinct subdiffusive CSLB dynamics inside the cluster and demonstrates the applicability of XPCS to low-concentration biologically relevant systems.

## Introduction

Synapsins are among the most abundant cytosolic proteins in the synapse and are critical for the assembly of the synaptic vesicle (SV) cluster (1,2,3). In solution, synapsin forms condensates by liquid-liquid phase separation (LLPS) and can recruit lipid vesicles (LVs) (2) or SVs into these condensates (3,4). As a model system, synapsin and vesicle condensates (either synapsin-SV or synapsin-LV) are considered to recapitulate the reserve pool of synaptic vesicles in the synapse (5). The neurobiology of synaptic vesicle clusters (SVCs) and the structure of in vitro models for the SVC has been relatively well studied by a number of techniques as reviewed in (6), ranging from cryoelectron microscopy (cryo-EM), to fluorescence light microscopy (7), to small-angle X-ray scattering (SAXS) (8). Contrarily, there is relatively little work on the dynamics within these condensates. Subdiffusive dynamics of synapsin in condensates was observed by single-molecule fluorescence (4). Regarding the vesicles, however, we currently ignore whether and how they diffuse within the cluster and how high the mobility is. Different regimes and scenarios seem possible, from the limiting case of arrested dynamics by a gel or network of synapsin, to corralled diffusion, or simply free diffusion with a reduced diffusion constant. More generally, biomolecular fluids are characterized by a complex interplay between structure, dynamics, and flow on a multitude of spatiotemporal scales (9). Viscoelastic properties and molecular mobility are of particular interest (for example, in protein networks of the cytoskeleton or in membrane assemblies). However, experiments are often complicated by the relevant length and timescales and the need for physiologically relevant environments.

We have recently explored passive X-ray microrheology to study the dynamics in biomolecular fluids, notably LLPS phases containing lipid vesicles (10). Similar to passive microrheology using optical microscopy, where the thermally driven motion of beads is recorded in complex fluids, X-ray experiments can be designed such that the signal is dominated by the scattering of tracer particles embedded into the fluid of interest. In contrast to optical microrheology, however, X-ray microrheology is carried out in reciprocal space. To gain insight into not only the structure but also the dynamics of the system, photon correlation spectroscopy is used, similar to dynamic light scattering (DLS). X-ray photon correlation spectroscopy (XPCS) can be regarded as an extension of DLS toward smaller length scales and to opaque or strongly scattering samples. As a first test system of passive X-ray microrheology or tracer-based XPCS, we previously used dense suspensions of LVs with added CaCl_2_. The strong nonlinear electrostatic interaction between anionic LVs and the divalent cations (11,12) resulted in adhesion of vesicles and formation of vesicles clusters. Since standard XPCS of unstained soft-matter systems often lack sufficient signal within the allowable dose budget to avoid radiation damage, colloids and nanoparticles are introduced into the sample to enhance contrast. Previous research has focused on either measuring the direct interactions between proteins and colloids, such as corona growth (13,14), or on passive X-ray microrheology with colloidal tracer particles in network-like phases (15,16). Here, we introduce colloidal tracers inserted into the protein-rich liquid phase to increase the scattering signal while indirectly probing dynamics of the sample solution or suspension in which they diffused. By boosting the SAXS signal and thereby the XPCS signal as a function of the scattering vector *q* as well, diffusion and viscoelastic properties of the biomolecular medium can be inferred. Importantly, this reduced the necessary dose to raise the photon correlation signals above background, a central challenge when studying biomolecular samples by XPCS. XPCS requires high coherence, offered by third- or fourth-generation synchrotron radiation (17) or X-ray free-electron laser radiation (18) as well as photon-counting pixel detectors with fast readout to cover the relevant timescales (19,20). Diffusion and transport modes of dense protein solutions have been studied in seminal XPCS experiments (21,22,23,24). However, the signal-dose relationship remains a central challenge (25), motivating the development of tracer-based passive X-ray microrheology. Note that such efforts are justified by the fact that dynamic observations with visible light also face severe limitations. Apart from the obvious example of opaque liquids, dense suspensions and multiple scattering, or in other cases issues of labeling and auto-fluorescence, impede many interesting applications. Further, the diffraction limit applies to conventional microscopy.

In tracer-based XPCS or passive X-ray microrheology, the interaction of the tracer particles with the phase to be studied presents a major concern. Silica nanoparticles, for example, can adsorb charged lipid vesicles (26). In practice, one relies on these interactions to be weak enough not to have a strong effect on the diffusion. A promising strategy seems to be to mask a colloidal tracer by enwrapping it with a lipid bilayer. This approach was used in (27) to study the interaction of vesicles and *α*-synuclein. They covered spherical silica nanoparticle by lipid bilayers (28) and used these colloid-supported lipid bilayers (CSLBs) in place of the vesicles to increase X-ray contrast in SAXS while retaining lipid-protein interactions and limiting interactions of the silica particles.

In this work, we adapt this approach to target the dynamical properties and in particular the diffusion of vesicles in synapsin-induced condensates or vesicle pools based on passive X-ray microrheology. To this end, we use lipid vesicles with a hard silica core, building on the protocol introduced in (28). We thereby boost the XPCS signal when probing dynamics in synapsin-induced condensates. The dynamics of the system exhibits aspects of both liquid-like and network-like behavior. Subdiffusive behavior is observed with effective-diffusion constants three orders of magnitude smaller than in pure buffer.

The manuscript is organized as follows: after this introduction, we begin by a description of the experimental methods and the CSLB protocol before discussing the main SAXS and XPCS results in the second part. The manuscript closes with a discussion that also summarizes the main conclusions.

## Materials and methods

### Vesicle production

For the preparation of LVs, lipids were purchased as powders from Avanti Polar Lipids (AL, USA) and dissolved in chloroform to yield 10-mg/mL stock solutions. Stock solutions of 1,2-di-(9Z-octadecenoyl)-sn-glycero-3-phosphocholine (DOPC), 1,2-di-(9Z-octadecenoyl)-sn-glycero-3-phospho-L-serine (DOPS), 1,2-di-(9Z-octadecenoyl)-sn-glycero-3-phosphoethanolamine (DOPE), and cholesterol were subsequently mixed to form a lipid composition of 55% mol DOPC, 15% mol DOPE, 20% mol DOPS, and 10% mol cholesterol, mimicking the lipid composition of SVs (29). Approximately 0.5% mol of Texas red-labeled DHPE (Thermo Fisher Scientific (MA, USA) was added for fluorescence microscopy. Lipid films were subsequently formed by evaporating the chloroform with a stream of nitrogen. To ensure complete removal of all solvent, lipid films were then dried in a vacuum chamber for >4 h before resuspension in a mix of acetate-buffered saline (ABS: 18 mM potassium acetate, 82 mM acetic acid, 150 mM NaCl, at pH 4.0) and 400 mM sucrose. Vesicles were then formed by freezing and thawing the solution in liquid nitrogen and a 37°C water bath 10 times. Finally, to obtain vesicles of similar size, they were pushed 21 times through a 50-nm polycarbonate membrane using a Mini Extruder (Avanti Polar Lipids).

### CSLB formation

**Figure 1:**
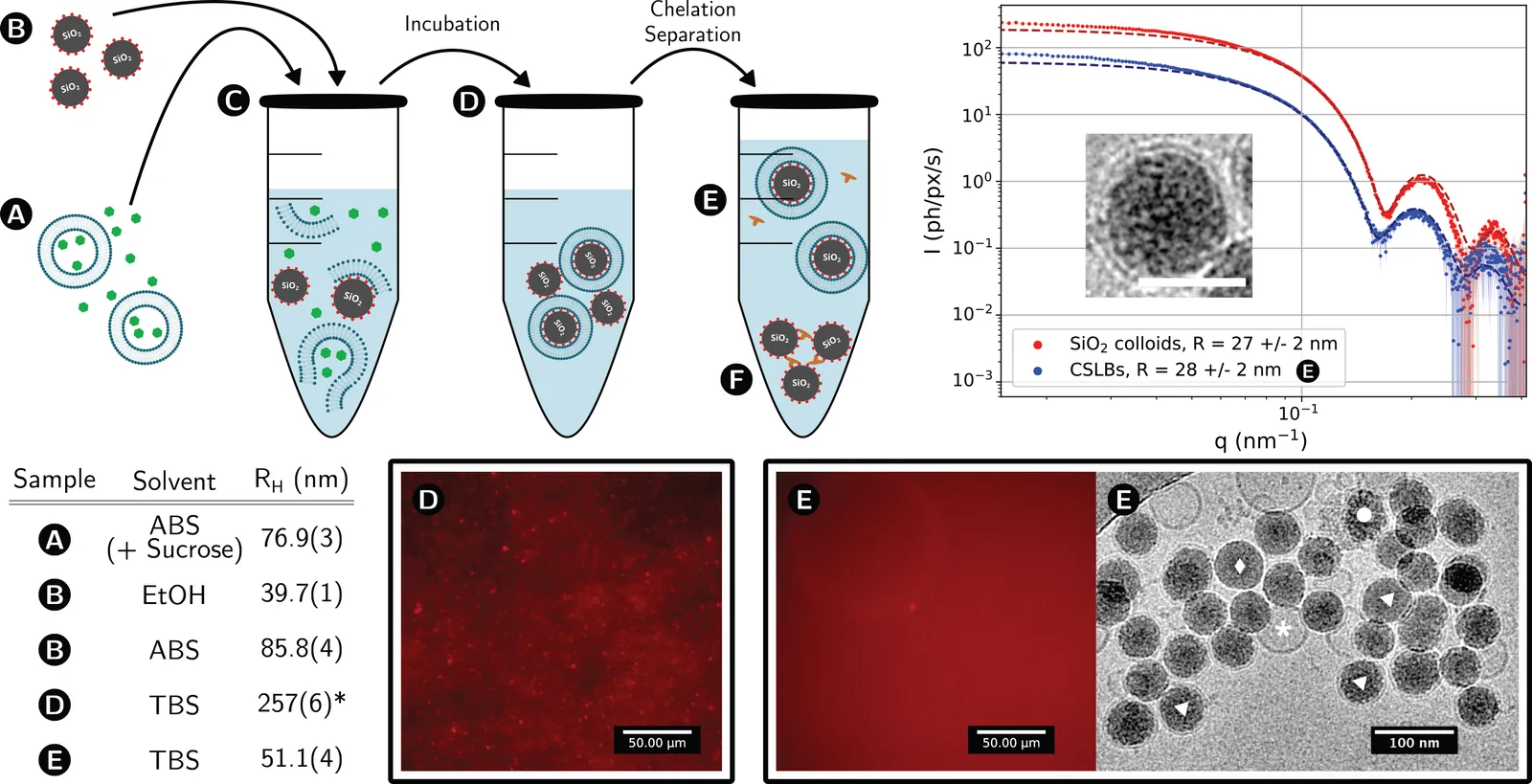
Protocol for CSLB formation and quality control of the obtained sample. CSLBs form after mixing lipid vesicles in high-sucrose buffer (*A*) and aminated silica colloids (*B*). A maximum yield is reached after incubation at 40°C (*D*). To separate coated and uncoated colloids, sodium citrate is added as a chelating agent (*orange prongs*). Aggregates of bare silica particles form after sonication (*F*), whereas the pure CSLBs remain monodisperse (*E*). Fluorescence microscopy of the sample before and after chelation (*D* and *E*, *bottom row center images*) serves as an initial test for the successful preparation. DLS measurements of the hydrodynamic radius *R*_*H*_ (table, asterisk [^∗^] symbol denotes strong aggregation, cf. supporting material) further show that the resulting CSLBs have a *R*_*H*_ similar to the initial colloid radius. Cryoelectron micrographs (*bottom right*) clearly reveal the coverage of colloids with lipid bilayers (*white triangles*) and also show that the coverage is not 100%, with some remaining vesicles (*stars*) and uncoated colloids (*diamonds*, *circles*). SAXS measurements (*top right*; data, dots; polydisperse spheres fit, dashed lines) show an increased average particle radius, further hinting at the presence of a lipid bilayer around a large percentage of the colloids. The inset shows a magnified cryo-EM image of a single CSLB with the bilayer clearly visible on the outside of the colloid (scale bar, 50 nm).

To form CSLBs of similar size to SVs, 50-nm-diameter aminated silica colloids were purchased from Nanocomposix (CA, USA). An overview of the protocol and the resulting CSLBs is given in Fig. 1. First, 500 *μ*L of the 10-mg/mL colloid suspension were centrifuged for 5 min at 14,000 rcf and placed under vacuum for 3 h after removal of the supernatant to obtain a dry stock of colloids. The dried colloids were then resuspended in acetate-buffered saline (without sucrose) at a concentration of 2 mg/mL and placed in an ultrasonic bath for 15, 5, and 5 min with thorough vortexing between each sonication step. The final colloid solution was checked for monodispersity and the absence of aggregation using DLS (ALV/CGS3 from ALV, Germany). A single exponential fit to the correlation function *g*^(2)^(*τ*), measured at 90° with a wavelength of *λ* = 632 nm, should yield a hydrodynamic radius *R*_*H*_ = 6*πηD*/*k*_*B*_*T* in the range of 80–90 nm (cf. sample B in Fig. 1). As an alternative to centrifuging and sonication, a dialysis was performed with a SpectraPor Micro Float-A-Lyzer (500 *μ*L, 50-kDa MWCO, Repligen, MA, USA) and pure acetate buffer without sodium chloride, yielding slightly smaller hydrodynamic radii. After the buffer exchange, 250 *μ*L of the colloids were thoroughly mixed with an equal amount of 30 mM LVs (pre-extrusion lipid concentration, sample A) and incubated for 1 h at 40°C and 500 rpm (sample C). Then, excess vesicles were removed and the buffer was exchanged for Tris-buffered saline (TBS: 25 mM Tris-HCl, 150 mM NaCl, 0.5 mM TCEP, at pH 7.4) by centrifuging the obtained solution at 1700 rcf for 15 min and subsequently replacing 450 *μ*L of the supernatant with TBS buffer. This process was repeated three times with thorough vortexing between each centrifugation step to redisperse the CSLB pellet (sample D). After letting the solution sit at 8°C for >8 h, the top 100 *μ*L were removed, mixed with 10 *μ*L of 100 mM sodium citrate dissolved in ultrapure water, and placed in a ultrasonic bath for 15 min to chelate any noncoated aminated silica colloids. Thus-formed silica aggregates (sample F) were separated from the CSLB solution (sample E) by a final step of centrifuging for 5 min at 100 rcf. The top 50 *μ*L of the supernatant constituted the final sample of CSLBs whose quality was again tested with DLS and should result in a hydrodynamic radius of *R*_*H*_ ≈ 50−60 nm. The final CSLB concentration was 0.31(3) mg/mL, determined by comparing SAXS intensities to a reference silica particle suspension of the same size (50 nm), as shown in the top right of Fig. 1 and detailed further in the supporting material. Additional characterizations with fluorescence microscopy and cryo-EM are shown in the bottom row of Fig. 1. The cryo-EM data, obtained as described earlier (7), show that the CSLBs are fully covered with a lipid bilayer but that the preparation is not entirely perfect, leaving some uncoated colloids and intact vesicles in the solution. However, the shift in the first minimum of the SAXS curve compared to the bare colloid sample and the corresponding increase in the particle radius indicates that the majority of the colloids are covered with a lipid bilayer. Furthermore, the single exponential decay of the DLS signal (cf. supporting material) and the flat Guinier region (*q* ≪ 0.1 nm^−1^) demonstrate that the resulting sample is indeed monodisperse and not aggregated.

### Protein expression and protein-vesicle samples

Human synapsin Ia was expressed from Expi293 cells (Thermo Fisher Scientific, MA, USA) and purified as previously described (2). The synapsin Ia stock solution contained 19.6 *μ*M of the protein in TBS buffer. Proteins were always handled on ice to reduce protein activity before mixing it in a sample. When not in use, they were flash frozen in liquid N_2_ and stored at −80°C. Protein and CSLB solutions were mixed at desired protein-to-lipid ratios (P/Ls), corresponding to spherical and dense condensates, i.e., at values in the phase diagram (7), where the P/L is higher than the transition between fractal and compact condensate morphology. The very low P/L ratios, which lead to visibly different, large fractal-like clusters, discussed in (7), were hence not investigated here. Specifically, we report data for three different samples at P/L ratios of 1:11, 1:6, and 1:3, with CSLB mass concentration of 0.2, 0.15, and 0.1 mg/mL, respectively.

### X-ray setup

The XPCS experiments were carried out at the ID10-COH beamline at the European Synchrotron Radiation Facility (ESRF) (30). A custom-built Eiger500k pixel detector (Dectris, Switzerland and PSI Detector Group) operating at a maximum frame rate of about 22 kHz was used for all XPCS measurements (31). High intensity X-rays are generated by an electron beam passing through three undulators located 61 m upstream from the sample position. The X-ray energy was set to 10.15 keV with a Si(111) channel-cut monochromator, and the beam dimensions were set by multiple sets of slits and beryllium lenses to approximately 30 × 30 *μ*m^2^ with a transverse coherence length of approximately the same dimensions. About 10 *μ*L of the sample was kept in a sealed 1-mm quartz capillary (Hilgenberg, Germany) placed on top of a Peltier element (set to *T* = 25°C, unless otherwise stated). The entire setup was kept inside the beamline vacuum, such that all measurements were taken fully in vacuum without intermediary windows. The photon-counting Eiger500k detector was located 5.38 m downstream from the sample. A set of attenuators placed about 1 m upstream from the sample were used to reduce the beam intensity on the sample. An overview of the main experimental parameters is given in the supporting saterial.

### Static X-ray analysis

The static scattering function *I*(*q*) was calculated directly from the summed 2D detector image of an XPCS train by azimuthal integration. SAXS data were subsequently normalized by exposure time and number of pixels per *q*-bin. The radiation damage inside the sample was monitored during the experiments by calculating the total dose *D* = (*μ*/*ρ*)*nt*_exp_*E*/*A* received by the sample during a measurement with the photon flux *n*, the photon energy *E*, the illuminated area *A*, and the exposure time *t*_exp_. The mass attenuation coefficient *μ*/*ρ* was assumed to be approximately that of water (32). Data were analyzed up to a dose limit of 200 kGy per XPCS train, beyond which significant changes with respect to the initial *I*(*q*) were observed. When longer trains were measured, the data were only considered up to this threshold. The radiation damage analysis was carried out as in (10), and is also further discussed in the supporting material, including an analysis of (possible) radiation-induced effect not only on the static structure but also on the dynamics (23).

### XPCS analysis

Each XPCS train comprised rapidly taking *N* frames with an exposure time *t*_exp_ at 10 % *I*_0_ (≈94 kGy/s). For fast measurements, 30,000 frames were taken at *Δt* = 50 *μ*s/frame and slow measurements were taken at *t*_exp_ = 1 ms/frame with *N* = 10,000 frames. Two frames were additionally separated by a detector latency time of 20 *μ*s (31), and each measurement position was separated from previous ones by at least 2 beam widths. The resulting set of 2D detector frames was subsequently processed using the *dynamix* software package (33). Processing consisted of calculating the azimuthally averaged scattering intensity *I*(*q*) and the intensity autocorrelation function *g*^(2)^(***q***, *τ*). The latter is calculated for entire regions *A*_*i*_ of the detector, because the beam intensity in a single pixel on the detector is too low to obtain a noise-free autocorrelation function per pixel *p*. In this case, the following formula is used to average over individual pixels *p* in a region *A*_*i*_:

(1) $$g^{\left(2\right)}\left(q_{i},\tau \right)=\frac{{\left\langle {\left\langle I\left(p,t\right)I\left(p,t+\tau \right)\right\rangle }_{p\in A_{i}}\right\rangle }_{t}}{{\left\langle {\left\langle I\left(p,t\right)\right\rangle }_{p\in A_{i}}{\left\langle I\left(p,t+\tau \right)\right\rangle }_{p\in A_{i}}\right\rangle }_{t}},$$

where *A*_*i*_ is a ring of radius *q*_*i*_ and width *Δq*, centered around the primary beam position *q* = 0   Before calculating the correlation functions, strong static flares are masked from the diffraction pattern. When a single XPCS train did not give a sufficient signal, a cyclic measurement scheme was used such that a correlation function could be obtained at a minimal dose rate on each measurement spot. Fast or slow XPCS trains with a maximum dose of about 200 kGy/train were recorded, separated by 0.1 mm along the *y* direction. Such a set of measurements was repeated up to 10 times on the same set of positions without any visible changes to the static scattering function that imply radiation damage (cf. supporting material). This allowed measuring the local dynamics beyond a total local dose of 200 kGy at a very low local dose rate, due to the long time between XPCS trains taken on the same spot.

## Results

**Figure 2:**
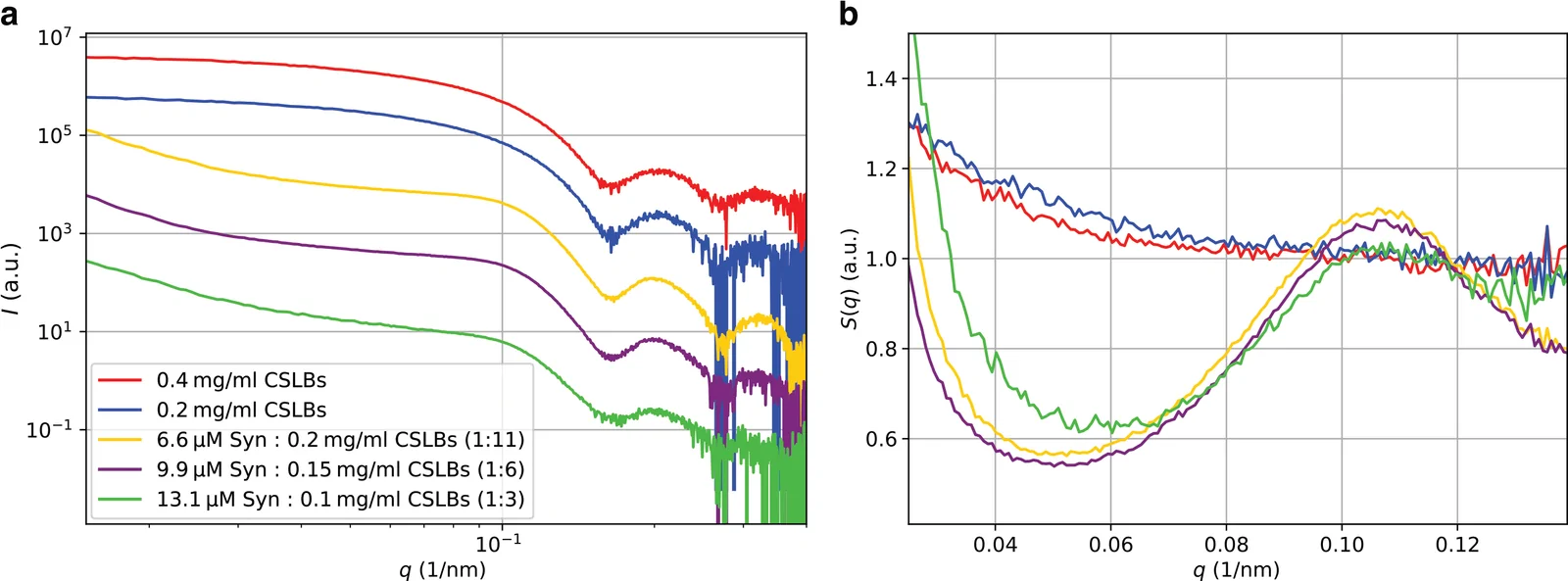
(*a*) Background-subtracted scattering curves *I*(*q*) of CSLB-protein mixtures of various concentrations and (*b*) corresponding normalized structure factor *S*(*q*). The structure factor was obtained from dividing the scattering curve by a fitted spherical form factor obtained from the dilute CSLB sample (*blue curve*). The pronounced increase of scattering intensity toward *q* → 0 is specific to samples containing synapsin protein (Syn). A second Syn-specific structure factor peak occurs at approximately 0.11 nm^−1^. The peak is less pronounced at the highest Syn concentration (*green curve*). At lower Syn concentrations, the structure factor peak appears to be much less sensitive to the Syn concentration. The scattering curves in (*a*) were shifted for clarity.

**Figure 3:**
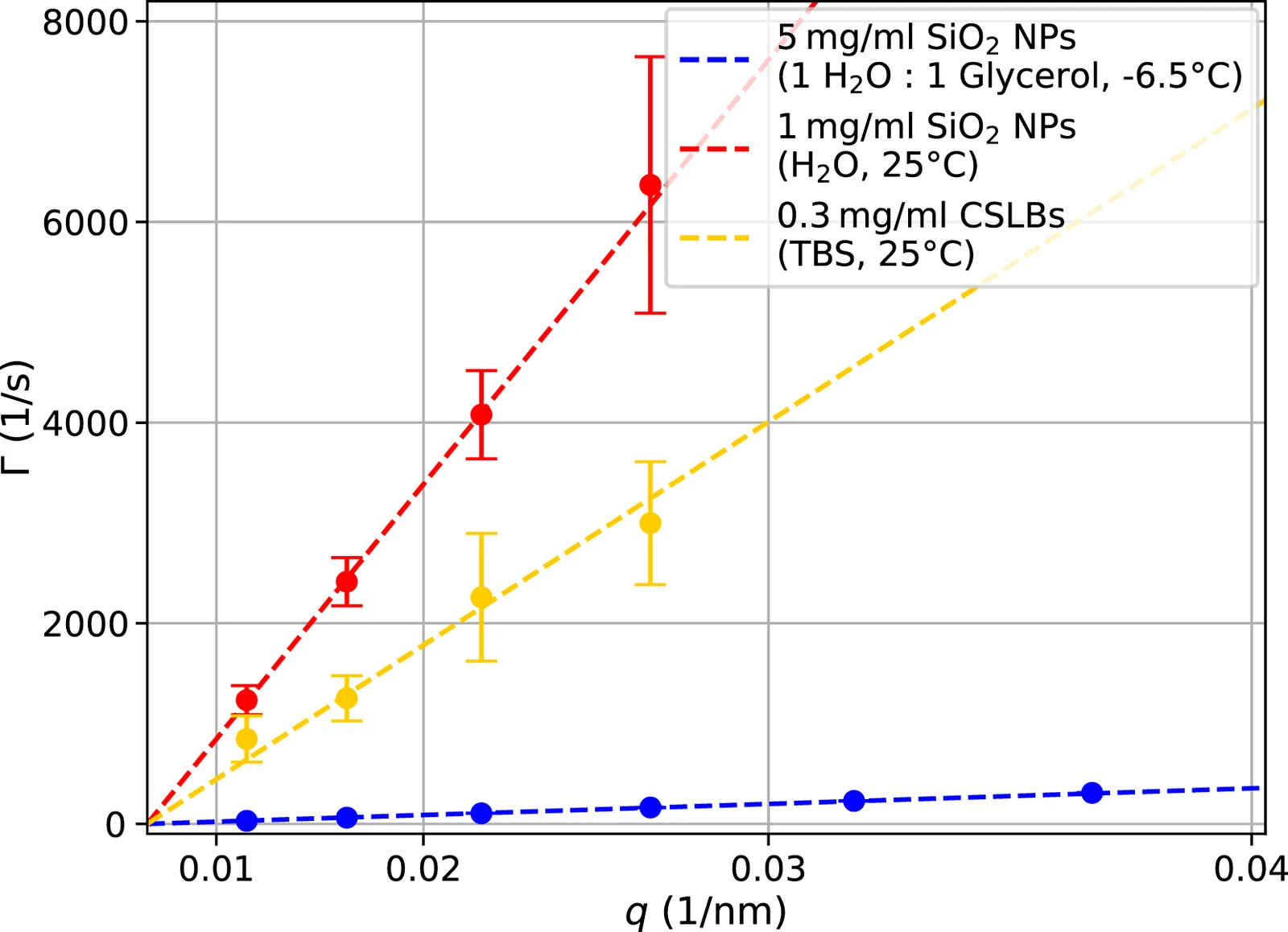
Relaxation rates *Γ*(*q*^2^) for freely diffusing silica particles and CSLBs measured by XPCS. The data (*circles*) and the corresponding errors were obtained from fits to the correlation functions. Quadratic fits were carried out to determine the diffusion coefficient and hydrodynamic radii *R*_*H*_ of each sample. The resulting parameters are tabulated in Table 1. Although the correlation functions of silica particles suspended in a glycerol-water mixture can be fully sampled owing to its high viscosity, relaxations of a more dilute suspension in water are much faster. As a result, larger deviations from the expected *R*_*H*_ of 26 nm occur, and the accessible *q* range is reduced. The quality of the fit for the CSLB sample at 0.3 mg/mL is further reduced, but clearly shows the same increased diffusion constant (reduced slope) that was also observed in the DLS measurements of the CSLB sample. The corresponding correlation functions are shown in the supporting material.

**Figure 4:**
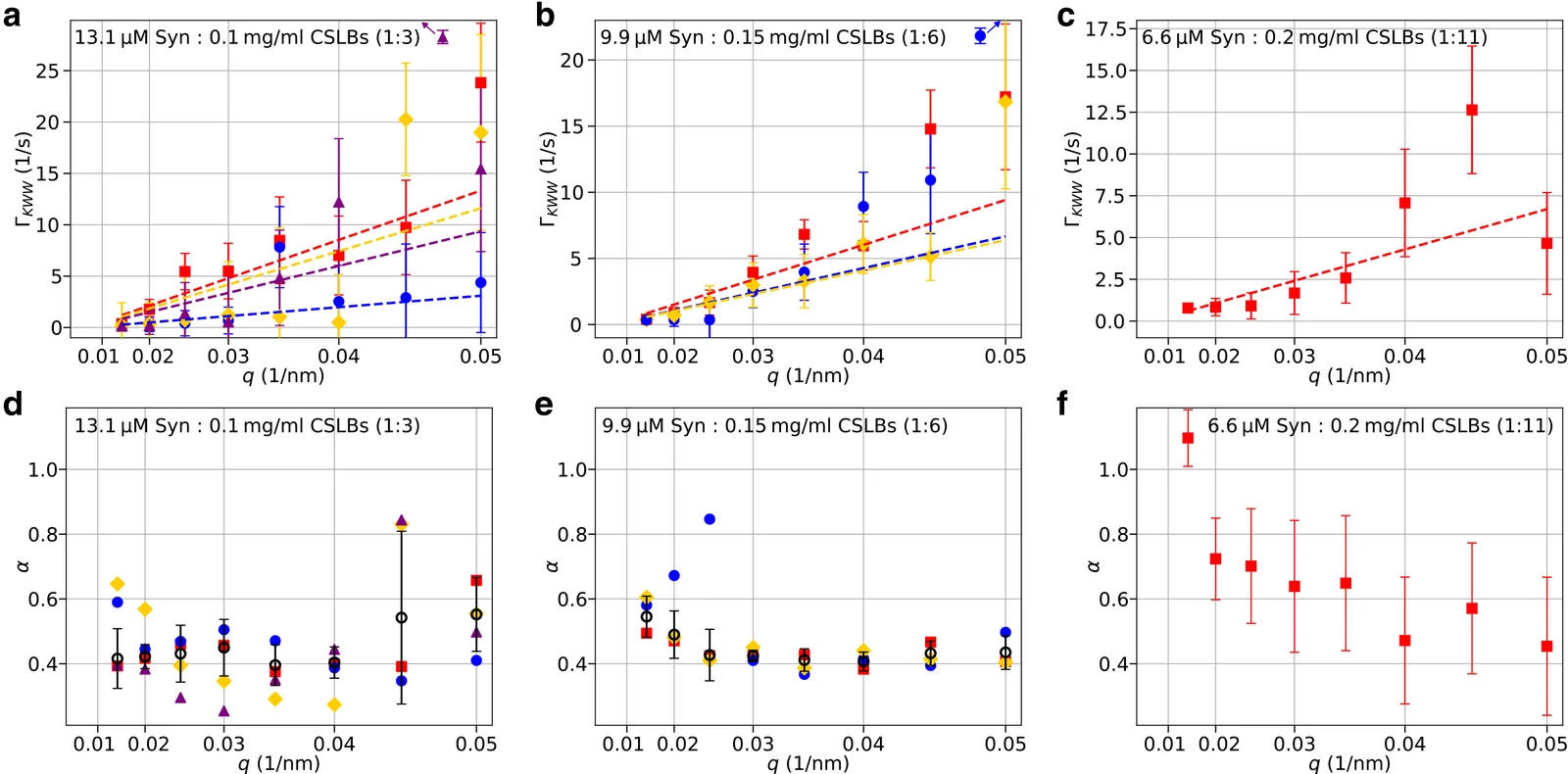
(*a*–*c*) Relaxation rates *Γ*_*KWW*_ and (*d*–*f*) corresponding KWW exponents *α* obtained from KWW fits to the XPCS measurements of samples with protein to lipid ratios (P/L) of (*a* and *d*) 1:3, (*b* and *e*) 1:6, and (*c* and *f*) 1:11. Different colors and symbols denote distinct measurement positions within each sample. Colored dashed lines in (*a*–*c*) show linear fits of the form *Γ*_*KWW*_(*q*^2^) = *D*_*eff*_*q*^2^ to each individual dataset. The resulting effective-diffusion constants *D*_*eff*_ are summarized in Table 2 together with an average ⟨*D*_*eff*_⟩ for each sample. Across all samples, the measured dynamics are strongly suppressed relative to freely diffusing CSLBs. The extracted KWW exponents indicate subdiffusive dynamics at approximately *α* ≈ 0.4 at high synapsin (Syn) content and display a lower positional variability than the relaxation rates. Weighted arithmetic means over the measurement positions are shown as black open circles and error bars in (*d*) and (*e*). At lower *q*, the sample with P/L ratio of 1:11 shows a trend toward standard diffusive behavior with values around *α* ≃ 1 (*f*). Data points with an arrow indicate a value outside of the visible plot range. The *x* axis is scaled quadratically for better visualization of the linear regression with respect to *q*^2^.

We first address the static SAXS results, presented in Fig. 2, followed by the dynamics in Figs. 3 and 4. The samples can be categorized into two classes: samples containing synapsin I (Syn) and CSLBs at various concentrations and control samples. The control samples consist of two samples of 50-nm silica colloid dissolved to 1 mg/mL in H_2_O and 5 mg/mL in a 1:1 mix of H_2_O and glycerol. Two further controls comprise CSLB samples in TBS buffer at two different concentrations (0.3 and 0.2 mg/mL). The protein samples contain synapsin protein and CSLBs at P/L ratios of 1:11 (0.2 mg/mL CSLBs:6.6 *μ*M Syn), 1:6 (0.15 mg/mL CSLBs:9.9 *μ*M Syn), and 1:3 (0.1 mg/mL CSLBs:13.1 *μ*M Syn). The synapsin-CSLB clusters were observed to sediment inside the capillary, which likely led to a higher absolute concentration of the two components at the measurement positions (bottom of horizontally oriented capillary). The ratio between the two components, which is key to the cluster-droplet transition (7), is, however, not affected by sedimentation.

### Structure of the synapsin-CSLB clusters

The structure of the synapsin-CSLB clusters was determined from an analysis of the structure factor *S*(*q*), calculated from the static SAXS measurements of the protein samples, as compared to the CSLB control samples. *S*(*q*) is extracted by separation of the form factor |*F*(*q*)|^2^ from the scattering intensity *I*(*q*). To this end, the scattering signal of a dilute suspension of CSLBs (*c*_*CSLB*_ = 0.2 mg/mL) is first fitted to the model function of polydisperse spheres given by

(2) $${\left|F_{\mathrm{fit}}\left(q\right)\right|}^{2}=\frac{a}{\sqrt{2\pi \Delta R^{2}}}\int_{-\infty }^{\infty }{\left(\frac{\sin\left(qR\right)-qR\cos\left(qR\right)}{{\left(qR\right)}^{3}}\right)}^{2}\times \exp\left(-\frac{{\left(R-R_{0}\right)}^{2}}{2\Delta R^{2}}\right)dR+b,$$

where *R*_0_ denotes the particle radius and *ΔR* the polydispersity. *a* and *b*, respectively, describe the intensity scaling and systematic offset (background) of the experimental scattering function with respect to the theoretical prediction, effectively scaling the form factor. To normalize each scattering curve separately, the form factor is then fitted again with *R*_0_, *ΔR*, and *b* fixed to the values obtained from the dilute suspension and *a* being the only free parameter. The full set of fit parameters is given in the supporting material. The structure factor of each sample containing CSLBs and protein is then obtained by division *S*(*q*) = *I*(*q*)/|*F*_*fit*_(*q*)|^2^ using the form factor from the dilute sample after scaling to fit *I*(*q*). The resulting *I*(*q*) curves are presented in Fig. 2 *a*. All samples clearly exhibit a spherical form factor, which dominates the signal at high *q*. At low *q*, an increase in the scattering intensity is observed for all samples containing synapsin, reflecting the presence of larger aggregates.

Fig. 2 *b* shows the corresponding structure factors for *q* ≲0.13 nm^−1^. Aside from the steep increase at low *q*, an additional structure factor peak is observed at around *q*_0_ = 0.11 nm^−1^, specific to samples containing synapsin. Its position corresponds to a length scale of 2*π*/*q*_0_ ≈ 57 nm, matching approximately the CSLB diameter. The two control samples do not exhibit any peak, indicating that this is a sign of CSLB-protein interactions and not due to clustering of the CSLBs itself. We also can note that the peak is strongest at low P/L ratios and less pronounced at a ratio of 1:3. From the presence of the interference peak, we can hence conclude that synapsin recruits CSLBs into condensates formed by LLPS as it does for lipid or SVs. From the peak position at *q*_0_, one can then obtain the maximum of the pair correlation function; i.e., the interparticle distance of CSLB colloids within the condensate as 2*π*/*q*_0_ (34). However, this relation is only valid for a compact liquid structure. In case of a fractal morphology, the structure factor shifts slightly to higher *q*, and the value 2*π*/*q*_0_ hence becomes only a lower bound for the interparticle distance (8). Note that a transition was previously observed for synapsin and vesicle phases by fluorescence light microscopy from spherical condensates at high P/L ratio to condensates of fractal appearance at low P/L (7).

### XPCS analysis

Next, we address the dynamics of CSLBs and synapsin, measured by XPCS. We first analyze the dynamics of the control samples to establish a reference for the dynamics of freely diffusing colloids and CSLBs. We then turn to the analysis of the samples containing synapsin, where we observe a much slower relaxation of the correlation functions, which we attribute to the diffusing particles inside the cluster.

### Free diffusion

Data were first recorded for freely diffusing particles to serve as controls for the synapsin-CSLB measurements. To this end XPCS trains were acquired (i.e., recordings with a pre-defined sequence of detector frames with selected sampling rate). For each XPCS train, the correlation functions *g*^(2)^(*q*, *t*) were analyzed for selected *q*-bins read out from the two-dimensional detector, and the relaxation rate *Γ* was determined by fitting a single exponential decay *g*^(2)^(*t*) = *b* + *β* exp(−*Γτ*) to the measured correlation function, where *b* determines the baseline and *β* the speckle contrast. A diffusion constant *D* was subsequently determined from a linear fit to *Γ*(*x*) = *xD*_*eff*_ with *x* = *q*^2^ and used to calculate the hydrodynamic radius *R*_*H*_ via the Stokes-Einstein relation. The results are shown in Fig. 3 and the corresponding fit parameters and results are tabulated in Table 1. Corresponding intensity correlation functions *g*^(2)^(*q*, *τ*) are shown in the supporting material. We begin with a determination of *R*_*H*_ for the pure SiO_2_ particles, which we measured at 5 mg/mL and −6.5°C in a 1:1 mixture of water and glycerol, to ensure optimal sampling and high accuracy. In fact, this enabled complete sampling of the full correlation function across a wide range of *q* values (see supporting material), yielding *D* = 0.220(2) *μ*m^2^/s and *R*_*H*_ = 26.0(2) nm with very low uncertainties. At one-fifth of the particle concentration and in pure water at room temperature, *D* is fitted to 8.50(7) *μ*m^2^/s with a relative error similar to the one of the measurement at high colloid concentration. This is obtained despite the inferior sampling of *g*^(2)^(*q*, *τ*) due to much faster dynamics, cutting off most of the correlation function and limiting the evaluation of values beyond *q* ≈ 0.03 nm^−1^ (see supporting material for correlation functions *g*^(2)^(*τ*, *q*)). Note that the 3-nm difference in *R*_*H*_ between the two controls likely originates from the different solvent or a viscosity slightly deviating from the literature values used in the determination of *R*_*H*_. Finally, we consider the signals from the CSLB sample, which are even weaker, due to the low particle concentration. The fits yield *D* = 4.50(7) *μ*m^2^/s, which is significantly slower than the pure colloids. The hydrodynamic radius, however, is determined to *R*_*H*_ = 55(4) nm, in good agreement with the radius determined earlier by DLS at better signal to noise ratio. The controls demonstrate that even fits to incompletely sampled correlation functions yield reasonable estimates for *D* when measurements at multiple *q* values are taken into account.

**Table 1:** Fitted Diffusion Constants *D* and Calculated Hydrodynamic Radii *R*_*H*_ of Three Colloid Control Samples

Sample	Solvent	*D* (*μ*m^2^/s)	*T* (°C)	*η*_*lit*_ (mPas)	*R* (nm)
5 mg/mL SiO_2_ NPs	1 H_2_O:1 glycerol	0.220(2)	−6.55	33.27	26.0(2)
1 mg/mL SiO_2_ NPs	H_2_O	8.50(7)	25.1	0.891	29.0(2)
0.3 mg/mL CSLBs	TBS	4.5(3)	25.1	0.891	55(4)

Diffusion constants are obtained from a linear regression of the relaxation rates *Γ*(*q*^2^) shown in Fig. 3. The temperature of the capillary was controlled with a Peltier element and the solvent viscosities were taken from literature: (35) for water and (36,37) for the glycerol-water mixture.

### Synapsin samples

We now turn to the samples containing mixtures of CSLBs and synapsin at various P/L ratios (1:3, 1:6, and 1:11). The XPCS data acquisition and analysis involved a number of different steps, owing to the weak scattering signal that required averaging of multiple measurements to obtain a sufficient quality of the correlation functions. The initial processing step of calculating the correlation functions is performed in two different *q* ranges for all XPCS trains. The first range covered values from the lowest accessible *q* up to 0.06 nm^−1^ with bin widths of 0.005 nm^−1^. The second range covered values from 0.05 to 0.11 nm^−1^, such that the highest *q* values analyzed included the location of the structure factor peak. To compensate for the lower signal at high *q*, the bin width was widened to 0.015 nm^−1^. Only a single XPCS train at a sampling rate of 1 kHz was captured up to the 200-kGy limit at a P/L of 1:11 (6.6 *μ*M Syn:0.2 mg/mL CSLBs). The other two samples, at P/L 1:6 and 1:3, were measured cyclically, as described above. The measurements were first separated by position and then averaged, after the observation that the dynamics at each measurement position are similar. Outliers, such as strong baseline variations, were removed before averaging. The correlation functions of all samples and measurement positions were subsequently fitted at each *q* value using a Kohlrausch-William-Watts (KWW) stretched exponential function of the form

(3) $$g_{\mathrm{KWW}}^{\left(2\right)}\left(\tau \right)=b+\beta \exp\left(-2{\left(\tau /\tau_{\mathrm{KWW}}\right)}^{\alpha }\right),$$

where the four fit parameters *b*, *β*, *τ*_*KWW*_ = 1/*Γ*_*KWW*_, and *α* denote the baseline, speckle contrast, relaxation time, and KWW exponent, respectively. The speckle contrast was bound to plausible values in the interval $\beta \in \left[0.028,0.033\right]$. The KWW-exponent *α* describes the functional form of the decay of the correlation function. At fixed decay time (relaxation time) higher *α* indicates a faster relaxation (decorrelation), whereas the function has a more pronounced tail for smaller *α*. In real space, this corresponds to the scaling of the mean-squared displacement with time (16) or, equivalently, the width of the distribution of the relaxation times present in a system (38). For normal free diffusion *α* = 1, whereas ballistic dynamics is associated with *α* = 2 (e.g., translation). More generally, *α* > 1.0 corresponds to superdiffusive motion and *α* < 1.0 implies slower dynamics than normal diffusion, indicating a broad distribution of different relaxation times. The correlation functions for each measurement position are shown for all samples in the supporting material. In general, the relaxation rates are reduced by more than three orders of magnitude compared to free CSLBs, indicating severely restricted dynamics in the presence of synapsin. Although the model fits become ambiguous at high *q* (*q* > 0.05 nm^−1^, cf. Fig. 4 of the supporting material), and suffer from low signal to noise, a linear relationship between *Γ*_*KWW*_ and *q*^2^ yields reasonable agreement in the low *q* region. An effective-diffusion constant *D*_*eff*_ can hence be obtained from linear fits to the model *Γ*_*KWW*_(*x*) = *xD*_*eff*_ with *x* = *q*^2^.

Fig. 4 shows the resulting values for the relaxation rate *Γ*_*KWW*_ (Fig. 4, *a*–*c*) and the KWW-exponent *α* (Fig. 4, *d*–*f*), for each sample and individual measurement position. To obtain a reasonable estimate for the average effective diffusion ⟨*D*_*eff*_⟩, we excluded obvious outliers and values of *D*_*eff*_ associated with a poor linearity of *Γ*(*q*^2^) (fit quality $R_{\mathrm{adj}}^{2}<0$), which indicates that a simple effective-diffusion description is not appropriate over the corresponding *q* range or for the local dynamics at those positions. Although all data originate from the same measurements, spatial heterogeneity leads to varying degrees of compatibility with the effective-diffusion approximation. Restricting the analysis to positions where this approximation provides a reasonable description allows the extraction of an average ⟨*D*_*eff*_⟩ that, although model-dependent, serves as a useful parameter to assess the dynamical slowing of the system. All measured positions, including those excluded due to poor fit quality, are reported in the supporting material together with their corresponding *D*_*eff*_ and $R_{\mathrm{adj}}^{2}$.

The individually fitted *D*_*eff*_ per sample and measurement position are tabulated in Table 2. For the samples with P/L 1:3 and 1:6, where more than one position was measured, fits were carried out separately for each measuring position. To then obtain an averaged effective diffusion for each sample, an average ⟨*D*_*eff*_⟩ was calculated over all positions per sample excluding obvious outliers and individual positions where $R_{\mathrm{adj}}^{2}<0$. The error of ⟨*D*_*eff*_⟩ was calculated as the standard deviation over different measurements $\sqrt{\left(\sum {\left(D_{\mathrm{eff}}-\left\langle D_{\mathrm{eff}}\right\rangle \right)}^{2}\right)/N}$. The variation of *D*_*eff*_ with measurement positions in the 1:3 sample reflects the challenge of low scattering intensity in this sample, due to the lower concentration of CSLBs. The resulting diffusion constants ⟨*D*_*eff*_⟩ range between 0.0027 and 0.0037 *μ*m^2^/s.

**Table 2:** Fit Results for Linear Fits to Relaxation Rates

Sample	*D*_*eff*_ (*μ*m^2^/s)	
	Positional	Average
1 Syn:3 lipid	0.0037(20)	0.0037(16)
	0.0053(10)	
	0.0012(5)	
	0.0046(16)	
1 Syn: 6lipid	0.0027(9)	0.0030(5)
	0.0026(4)	
	0.0038(7)	
1 Syn:11 lipid	–	0.0027(5)

Tabulated values correspond to the fits of the relaxation rates shown in Fig. 4, *a–c*. A linear fit function *f*(*q*^2^) = *D*_*eff*_*q*^2^ was used where the slope corresponds to the effective-diffusion constant *D*_*eff*_. The CSLB dynamics slow down markedly in the presence of synapsin (Syn). *D*_*eff*_ varies for different measurement positions, in particular at low P/L where the scattering intensity was much lower and noisy measurements were compensated for by several repeats at different positions. Corresponding fits to the correlation data are shown in the supporting material.

The data further support a subdiffusive scaling of the dynamics (*α* < 1). In fact, most values for the KWW-exponent *α* are distributed around approximately *α* ≈ 0.4 for the first two samples with P/L ratios of 1:3 and 1:6, whereas the sample with lowest P/L ratio of 1:11, shows a *q*-dependent decay from *α* ≈ 1.0 at *q* ≤ 0.035 nm^−1^ to *α* ≈ 0.4 at *q* ≈ 0.04 nm^−1^ and onward. The low values of *α*, corresponding to stretched exponentials, indicate a broad distribution of relaxation times, often observed near a glass transition (25,39). The higher KWW exponent at low *q* in the sample of the lowest P/L ratio suggests that the diffusive motion of the CSLBs is less impacted when less synapsin is present. The fit uncertainties and low number of samples do not allow us to draw detailed conclusions regarding the precise dependence of the dynamics on P/L.

In addition to the relaxation discussed above, we also observed a second decay at long timescales O(10s), which scaled only very weakly or not at all with *q*. The KWW exponent indicated diffusive or slightly superdiffusive behavior, which we attribute to the relaxation to some form of external motion. This conclusion is corroborated by earlier simulations of a toy model investigating the rotation and shear of particles, mimicking tumbling clusters of particles, where similar dynamics were observed with rotating clusters (10). Further details on this relaxation are provided in the supporting material.

## Discussion

Synapsin I is well known to induce a liquid-liquid phase transition (2) and to condense lipid vesicles into clusters (7). The primary goal of this work was to quantify the dynamics of lipid vesicles in these condensates. Without contrast enhancement, XPCS microrheology suffers from low scattering intensity, and the sensitivity to radiation damage impedes accumulation of signal by prolonged acquisition times (10). Here, we therefore inserted inorganic colloidal tracers that yield stronger scattering signal. To ensure that the tracers did not perturb the system under study, we used CSLBs to mimic the lipid vesicles. At the same time, the silica core of the particles ensured a signal high enough to calculate correlation functions within the allowable dose budget. Pure CSLBs in buffer did not show any pronounced modulation or peak in the structure factor; i.e., the colloids did not aggregate/form condensates. Contrarily, a peak at *q*_0_ ≈ 0.11 nm^−1^ appeared in all samples containing synapsin, reflecting interparticle correlations induced by the protein. The peak corresponds to an interparticle distance of approximately 2*π*/*q*_0_ ≈ 57 nm, assuming the standard relation of a compact liquid. In addition to the peak, the structure factor showed an increase for *q* → 0, indicative for liquid-liquid phase separations and the formation of condensates.

XPCS on these samples revealed a significant slowing down of the colloid dynamics. Although the XPCS measurement on dilute CSLBs suspended in buffer yielded a diffusion constant of *D* = 4.5(3) *μ*m^2^/s, which is in good agreement with the theoretical prediction of freely diffusing spheres, a significant retardation of the dynamics was observed in the presence of synapsin. Averaging over multiple XPCS trains resulted in effective-diffusion constants in the range of ⟨*D*_*eff*_⟩ = 2.7 × 10^−3^ to 3.7 × 10^−3^ *μ*m^2^/s. These findings suggest that the CSLBs can indeed move inside synapsin condensates but at a very reduced rate compared to the case of free diffusion in a buffer solution. The similar *D*_*eff*_ values for all P/L and the KWW exponent *α* ≈ 0.4, which was also observed for all measured synapsin samples at high *q*, indicates that the type of dynamics does not change significantly with P/L. This suggests the conclusion that the interactions remain similar, whereas the density of the environment changes, a result that is in line with the conclusions drawn from static scattering. The presented results are likely to translate to lipid vesicles, as the protein will interact similarly with vesicles and with CSLBs due to the lipid coverage of the colloids. Lipid vesicle deformation, reported for Syn-LV interactions but absent in Syn-SV systems (7), is, however, not accessible within the CSLB model. The fact that CSLBs still cluster under the influence of synapsin indicates that the elastic deformations of vesicles do not play a crucial role in synapsin pool formation. Furthermore, we speculate that the pronounced subdiffusive behavior with *α* < 1, which is observed here for the multivalent interactions between synapsin and vesicles, could possibly be a hallmark of phase separating biomolecular fluids.

Our results fit well into the context of earlier studies on protein mobility inside SynI-LV condensates, where the subdiffusive dynamics of individual molecules was measured (4). The scaling law (*α* ≈ 0.5) and effective diffusion (*D*_*eff*_ ≈ 0.003 *μ*m^2^/s) reported in that work are similar to the values found here. Hence, the mobility of vesicles inside the condensate may be limited by the same interactions as for the protein itself. The similar effective-diffusion coefficient of the two components suggests that synapsin does not just provide an environment in which the vesicles are suspended but that both components may move together. Alternatively, the interaction with a synapsin network, possibly with transient bonds constantly assembling and disassembling, may determine the dynamics inside the condensate. We can conclude that the condensates are definitely not rigid structures. At the same time, they differ from a liquid state where vesicles move with simply a rescaled diffusion, since in that case we would have *α* ≈ 1. It rather seems that the condensates represent a viscoelastic medium intermediate between liquid and solid. In line with (4), where fluorescently labeled synapsin was tracked in condensates, a network-like structure induced by synapsin could be a plausible explanation for the subdiffusive behavior. This could then also account for various factors that influence the protein-lipid interaction and indirectly also the transport properties, such as P/L ratio, phosphorylation, and overall concentration. Unfortunately, we lack a model to make such an explanation quantitative and to test it against the observed dynamics.

To conclude from a methodological point of view, we have shown that XPCS microrheology is a promising solution to access the local dynamics of synapsin-vesicle clusters. However, tight limits on the applied dose and necessary scattering intensity require careful measurements and subsequent analyses to extract reliable results. Here, cyclic measurements that minimized sample exposure were found to provide sufficient signal, even though variations in the local dynamics can complicate the later averaging of correlation functions or fit parameters.

## Data and code availability

• All XPCS and SAXS data underlying this work have been collected under the proposal of SC-5574 “Dynamics of vesicles in dense pools studied by XPCS-microrheology (cont’d),” and are publicly available under at the Database ESRF data portal (https://data.esrf.fr) under https://doi.org/10.15151/ESRF-ES-1690152590.

## Acknowledgments

We thank Arsen Petrovic and Rubén Fernández-Busnadiego for help with cryo-EM and a related collaboration. This work was funded by the 10.13039/501100001659Deutsche Forschungsgemeinschaft – SFB1286 Quantitative Synaptology, Projects A2 and B10. We thank the ID 10 for the provision of beamtime and synchrotron radiation facilities. We thank the Partnership for Soft Condensed Matter, in particular Pierre Lloria and Diego Pontoni, for access and support to their laboratories at the ESRF. H.B. acknowledges funding from 10.13039/501100001659Deutsche Forschungsgemeinschaft – Project-ID 449750155 – RTG 2756, Project B2.

## Author contributions

T.C. and T.S. designed research. T.C. and A.M. carried out sample preparation. T.C., A.M., H.B., M.C., and T.S. carried out the experiments. T.C. analyzed data. C.H. and D.M. purified the proteins and provided expert advice on the system. T.C. and T.S. wrote the manuscript.

## Declaration of interests

The author declares no competing interests.

## Supporting Material

Document S1. Figures S1–S11 and Tables S1 and S2

Document S2. Article plus Supporting Material

## Footnotes

Supporting Material can be found online at https://doi.org/10.1016/j.bpj.2026.03.006.
