# Supplementary material for: Vesicle dynamics in synapsin-induced condensates by passive X-ray microrheology
Source: Biophys J. 2026 Mar 6;125(7):1713–22. doi: 10.1016/j.bpj.2026.03.006 (PMC13351967; doi:10.1016/j.bpj.2026.03.006)
Supplement: Document S1. Figures S1–S11 and Tables S1 and S2 [file mmc1.pdf]

Biophysical Journal, Volume *125*

Supplemental information

Vesicle dynamics in synapsin-induced condensates by passive X-ray microrheology

Titus Czajka, Andras Major, Hendrik Bruns, Marco Cammarata, Christian Hoffmann, Dragomir Milovanovic, and Tim Salditt

Manuscript submitted to **Biophysical** *Journal*
Supporting Material

Vesicle dynamics in synapsin-induced condensates by passive X-ray microrheology

Titus S. Czajka[1], Andras Major[1], Hendrik Bruns[1], Marco Cammarata[2], Christian Hoffmann[3], Dragomir Milovanovic[3,4], and Tim Salditt[1,*]

[1]Institute for X-ray physics, Friedrich-Hund-Platz 1, Göttingen, 37077, Lower Saxony, Germany
[2]ESRF - European Synchrotron Radiation Facility, 71 Avenue des Martyrs, Grenoble, 38000, Rhone-Alpes, France
[3]DZNE - German Center for Neurodegenerative Diseases, Virchowweg 6, Berlin, 10117, Germany
[4]Institute of Biochemistry, Charité-Universitätsmedizin Berlin, Corporate Member of Freie Universität Berlin, Humboldt-Universität Berlin, and Berlin Institute of Health, Berlin, Germany
[*]Correspondence: tsaldit@gwdg.de

Table 1: Overview over the important beamline parameters used at the coherence branch of the ID10 at the ESRF.

Parameter	Symbol	Value	Source
Filling mode		Uniform @200 mA	
Energy	E	10.15 keV	
Monochromaticity	$\Delta\lambda/\lambda$	1.4×10^{-4}	(1)
Flux	n	1.2×10^{12} ph/s	
Beam size	A	30×30 µm	
Transverse coherence length	$l_{\perp}$	$20 - 40$ µm	(2)
Capillary diameter	x_{cap}	1 mm	
Capillary wall thickness	w	0.01 mm	
Distance sample-detector	d	5.38 m	
Detector type		Eiger500k CdTe	(3)
Detector pixel size	s_{px}	75 µm	(3)
Detector maximum frame rate	ν_{det}	22 kHz	(3)
Detector frame delay time	t_{gap}	20 µs	(3)

RADIATION DAMAGE

The challenge with XPCS measurements on dilute biological samples without damage is twofold: To correlate the individual speckle patterns, each frame has to have a minimum signal strength, which requires exposing the sample to a high dose rate, inflicting damage to the sample earlier (4). This implies that only short measurements are possible on sensitive samples, blocking access to slow sample dynamics. Measuring fast, on the other hand, requires short exposure times, which might not lead to a sufficient number of photons on the detector to calculate a correlation function. Translating the capillary might not always suffice, as the damage can spread along the capillary in long measurements (5, 6).

To keep the effects of radiation damage under control, we designed the measurements in accordance with our previous results, that indicated samples containing SynIa and vesicles can absorb a dose of approximately 200 kGy without showing signs of damage (5)[1]. This was checked by calculating the relative change of the scattering intensity

$$\Delta I(q,t) = \frac{I(q,t) - I(q,t_0)}{I(q,t_0)}\ , \tag{1}$$

where $I(q,t)$ denotes the scattering intensity at time t. The resulting evolution of $\Delta I(q,t)$ is shown together with the evolution of $I(q,t)$ in Figure 1a on a representative plot for a long measurement on a protein sample. 100 detector frames were binned for each row shown in the top part and for each graph drawn in the bottom part of Figure 1a to increase the signal to noise ratio at low exposure times. The total dose received by the sample at any point in time t is estimated using the equation mentioned in the main text, with $\mu/\rho = 5\,\mathrm{cm}^2/\mathrm{g}$ based on a linear interpolation of the values given in (7). The other parameters are taken from Table 1. The other measured samples behaved very similar to the two cases presented here. The Figure shows that even though the changes remain within 5-10 % of the total scattering intensity and are thus not visible in the absolute scattering curve, an increase in the relative intensity appears after about 200 kGy, indicating damage to the sample. This threshold is in agreement with the findings from a measurement without CSLBs, shown in Figure 1b. Note, however, that a significantly increased lipid concentration was necessary to obtain a signal above the background, changing the P/L ratio to approximately 1:12000.

The cyclic measurements do not show significant trends such as these over time, as we specifically designed the experiments to meet the 200 kGy criterion. The comparison between different iterations is shown in Figure 2, which compares the scattering intensities of the first 100 frames of all XPCS-trains at each position (colours indicate position). The scattering curves at each of the five positions visibly fluctuate around the corresponding initial measurement, indicating that the trend observed above is not present here. This suggests that the sample is not damaged significantly, because the effective dose rate at any position is greatly reduced by the time it takes to take measurements at all other positions.

Note that even if the scattering intensity does not change during the measurement, an effect of the beam on the dynamics of the system cannot be excluded (8). An analysis on how the decorrelation time changes with dose and/or dose rate is hence required to assess the effect of the beam on the measured samples for sensitive samples. To this end, we have evaluated the dynamical properties of the sample with the P/L ratio 1:11 for two different frame lengths, and hence dose. While 2000 frames (approx. 200 kGy) were considered tolerable in dose, 10000 frames (approx. 900 kGy) showed moderate changes, see Figure 3. The resulting fit parameters also only show small changes at 10000 frames with respect to the lower frame number, as shown in 4, vindicating the chosen parameters.

[1]The limiting dose mentioned in the cited paper is 30 kGy due to an error in the calculation.

SYNAPSIN CLUSTER XPCS – HIGH Q-RANGE

Correlation data was not only computed in the low-q range ($q \leq 0.06\,\mathrm{nm}^{-1}$), corresponding to length scales larger than the inter-vesicle distance, but also in the high-q range (0.05 to $0.11\,\mathrm{nm}^{-1}$), around the location of the structure factor observed in the static SAXS measurement. Due to the low signal to noise ratio at low CSLB concentrations, meaningful data in this range could only be obtained for the sample at P/L ratio 1:11. To compensate for the lower signal at high q, the bin width is increased from $0.005\,\mathrm{nm}^{-1}$ (low-q range) to $0.015\,\mathrm{nm}^{-1}$ (high q-range). Correlation functions were computed from 2000 frames captured at a frame rate of 1 kHz, giving a total measurement time of 10 s and a total dose of approximately 200 kGy. Each correlation function was subsequently fitted using a Kohlrausch-William-Watts (KWW) stretched exponential function

$$g^{(2)}_{KWW}(\tau) = b + \beta \exp\left(-2(\tau/\tau_{KWW})^{\alpha}\right) \ , \tag{2}$$

where the four fit parameters b, β and $\tau_{KWW} = 1/\Gamma_{KWW}$ model the baseline, speckle contrast, relaxation time and the KWW-exponent, respectively. The results are shown in Figure 3d and corresponding fit parameters are given in Figure 4a-d. Note that in the high q-range the dynamics becomes too fast for the plateau to be captured given the temporal sampling limit of the detection system. However, when setting fit bounds on β, information can still be extracted. This data shows that it would be very worthwhile to increase the detector frame rate, since the signal-to-noise is still reasonable in this very relevant regime, where dynamics can be probed on the characteristic length scales of the system.

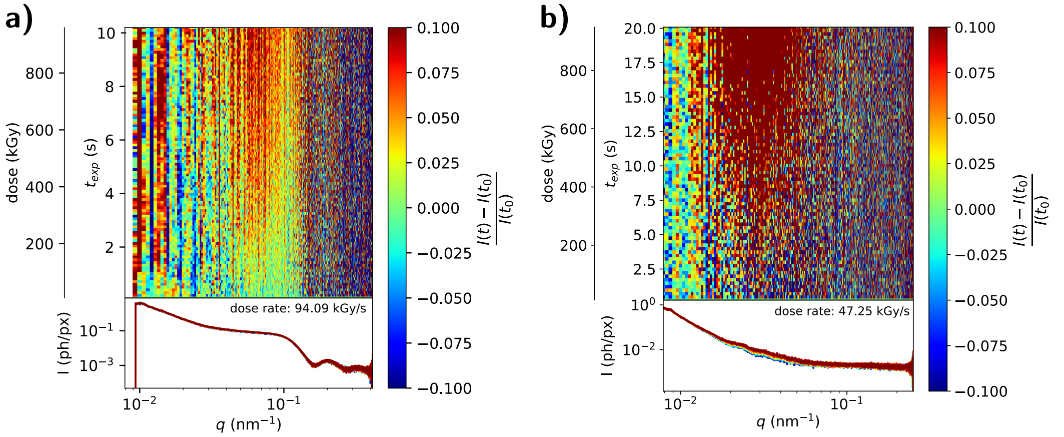

Figure 1: Radiation damage to a sample containing (a) SynI protein and CLSBs with P/L ratio 1:11 and (b) a similar sample with SynI and 36 mM lipid vesicles instead of CSLBs (P/L approx. 1:12000). The top part shows the change in scattering intensity for an average of 100 XPCS frames (= 100 ms) per row, relative to the first average. The bottom part shows the scattering intensity for each bin, from blue (first bin) to red (last bin). In (a), the beam-induced changes in the scattering signal (bottom) are only minute, even though visible in terms of the small relative changes (top), which are observed at a dose exceeding 200 kGy. Note that the beam-induced changes in the sample appear minute because they are screened by the radiation-hard structure of the silica beads which dominate the signal. This is different in the colloid-free sample in (b), where radiation damage is more directly visible in the scattering signal (bottom), see the splitting of curves around $q \approx 0.03\,\mathrm{nm}^{-1}$. Independent of the presence of colloids in the sample, we can conclude that damage starts to appear at a dose of approximately 200 kGy.

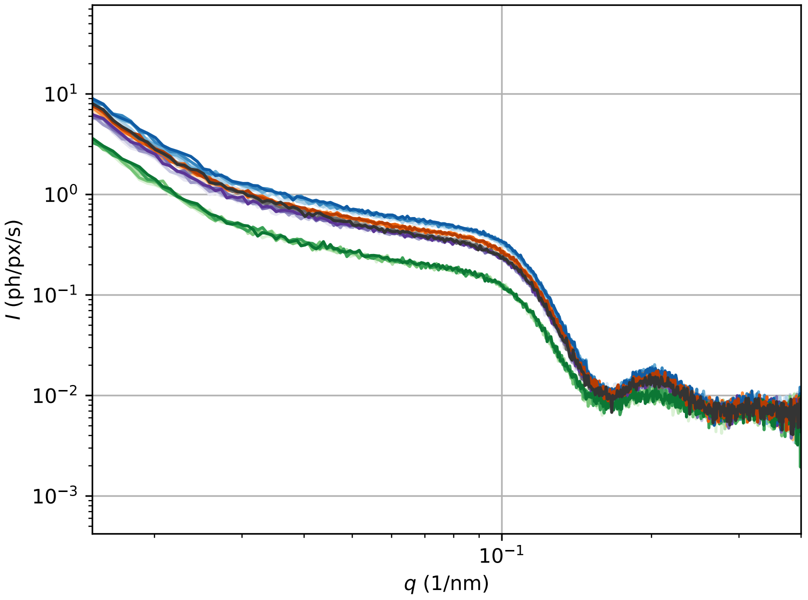

Figure 2: Changes in scattering intensity during a cyclic measurement of 5 cycles á 5 positions on the sample at P/L ratio 1:6. Different colours (grey, orange, blue, purple, green) represent the different positions along the cycle and the intensity of the colour indicates the iteration (from light to dark). At each position, no significant change in the intensity is visible. The sample appears equilibrated and undamaged by radiation. Different measurement positions can be distinguished clearly from each other, indicating an inhomogeneous sample distribution along the scanning direction. The shape of $I(q)$, however, remains similar at all positions.

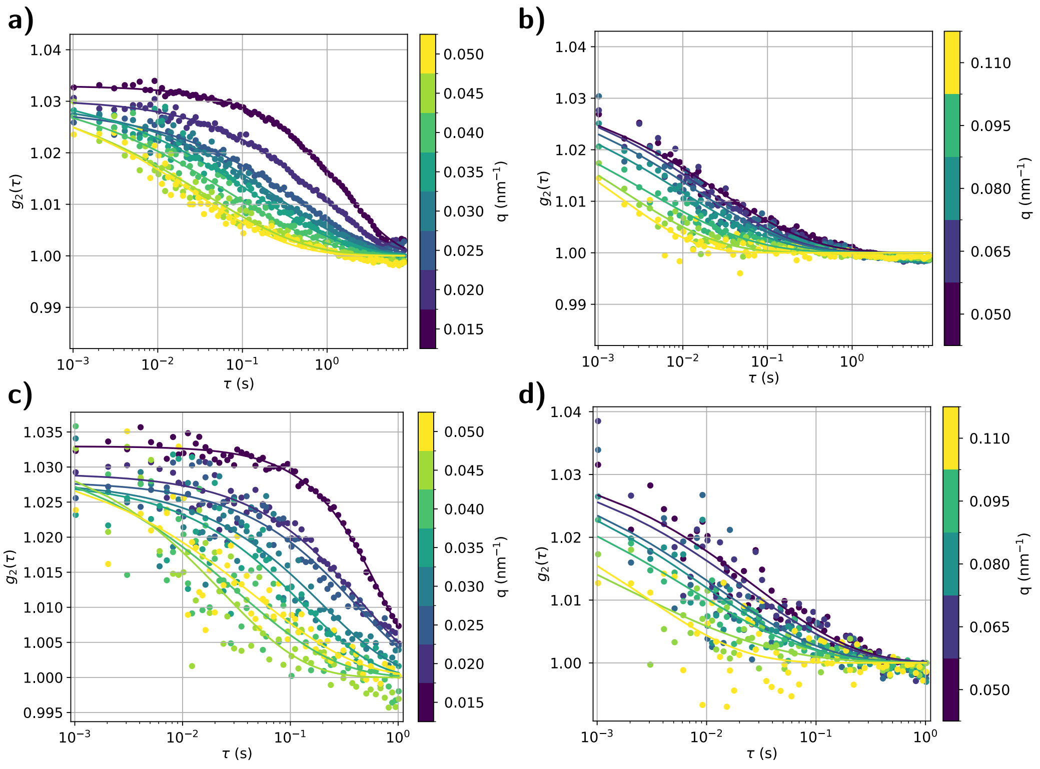

Figure 3: Correlation functions and corresponding least-square fits for the sample at P/L = 1 : 11 using a KWW fit function at two different values of accumulated dose. (a,b) Correlations calculated for 10000 frames (ca. 900 kGy) in (a) the low, narrow q-bins and (b) the higher, wider q-bins, respectively. (c,d) Same as (a,b) but only evaluated for the first 2000 frames (approx. 200 kGy). Moderate differences between the two correlation times arise, especially at low q values. A comparison of the obtained fit parameters is given in Figure 4. The larger bin width chosen for (b) and (d) result in an improvement in signal-to-noise. This allows an analysis of $g^{(2)}(q,\tau)$ up to the position of the structure factor peak at $q \approx 0.1\,\mathrm{nm}^{-1}$.

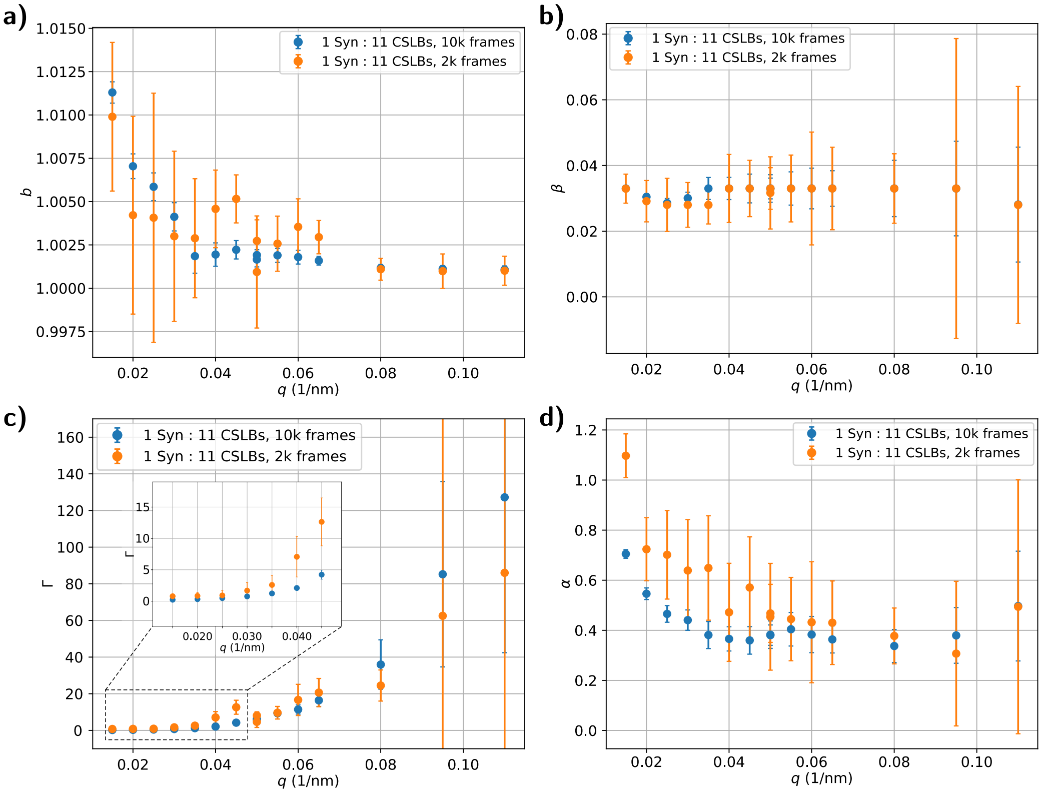

Figure 4: Parameters of the least-square-fit of the KWW-law to $g^{(2)}(q,\tau)$ with respective error bars across the full q-range, and for two values of accumulated dose, corresponding to the correlation data shown in Figure 3. Blue data correspond to the full XPCS-train of 10000 frames (ca. 900 kGy) while orange data points correspond to the reduced train of only 2000 frames (ca. 200 kGy). The parameters baseline b and contrast β displayed in (a) and (b), respectively, show no significant differences between low and high dose. The relaxation rate Γ shown in (c) decreases at higher dose, but the changes are mostly within the error margin of the fit. The KWW-exponent α shown in (d) exhibits more significant differences: the radiation affects the dynamics in a way which leads to a reduction in α at higher dose.

COLLOID SUPPORTED LIPID BILAYERS

To determine an approximate CSLB concentration, we compared a measurement at an unknown concentration c_{CSLB} with a measurement on a sample of known particle concentration c_{col} that has the same structure factors $S_X(q)$ and form factors $F_X(q)$ as $q \to 0$. Figure 6a illustrates this comparison for a sample of CSLBs at unknown concentration and a sample containing similarly sized SiO_2 colloids at 1 mg/ml. Assuming that lipids do not contribute significantly to the scattering signal due to their comparatively small electron density and that the background scattering from the buffer of both samples is similar (Figure 6b), we can set $\Delta\rho_{col} = \Delta\rho_{CSLB} = \Delta\rho_{\mathrm{SiO_2}}$. We can extract the unknown concentration c_{CSLB} from the ratio

$$\lim_{q\to 0} \frac{I_{col}(q)}{I_{CSLB}(q)} = \frac{V_{col}^2 N_{col}}{V_{CSLB}^2 N_{CSLB}} = \frac{V_{col} c_{col}}{V_{CSLB} c_{CSLB}} = \frac{R_{col}^3 c_{col}}{R_{CSLB}^3 c_{CSLB}} \tag{3}$$

and the relation $N_X = V_{tot} c_X / \rho V_X$, where V_{tot} denotes the total scattering volume and $V_X = \frac{4}{3}\pi R_X$ the volume of a single particle of radius R_X in sample X.

R_X can be obtained from fitting the form factor of polydisperse spheres to the scattering intensity. The form factor of monodisperse spheres can be calculated analytically (9) and the polydispersity is modelled assuming a normal distribution of particle radii around a mean radius R with standard deviation ΔR. Variations in the total scattering intensity are taken into account by a prefactor a. Additionally, a constant background b is assumed to take the signal-to-noise ratio of the measurement into account. Taken together, we obtain an expression for a fit function of four parameters (a, b, R_0, ΔR)

$$\left|F_{fit}(q)\right|^2 = \frac{a}{\sqrt{2\pi\Delta R^2}} \int_{-\infty}^{\infty} \left(\frac{\sin(qR) - qR\cos(qR)}{(qR)^3}\right)^2 \exp\left(-\frac{(R-R_0)^2}{2\Delta R^2}\right) \mathrm{d}R + b\ , \tag{4}$$

where the fit is performed as a least-squares optimisation with the integral calculated numerically for each iteration of the fit. Fits yield $R_{col} = 27(2)$ nm and $R_{CSLB} = 28(2)$ nm, where the uncertainty is taken to be the polydispersity ΔR. The obtained R_{col} are in good agreement with the datasheet of the colloids, which reports $R = 26(3)$ nm. The slightly increased radius of R_{CSLB} might hint at the presence of a lipid bilayer around the colloids.

We finally obtain the density estimate by rearranging Equation 3, which yields $c_{col}/c_{CSLB} = I_{col}(0) R_{CSLB}^3 / I_{CSLB}(0) R_{col}^3 \approx 3.25$ and we obtain $c_{CSLB} \approx c_{col}/3.25 = 0.31$ mg/ml. The uncertainties of this value are assumed to be dominated by variations of the scattering intensity of two measurements. The comparison between two background measurements in Figure 6b provides a reasonable estimate of this influence, giving an error of approximately 10 %. We thus obtain $c_{CSLB} = 0.31(3)$ mg/ml.

Table 2: Overview of the fit parameters and quality of fit for the colloid and CSLB SAXS data used to determine the CSLB density (SiO_2 and 0.3 mg/ml CSLBs). Additionally, the same values are tabulated for the CSLB measurement that was used to calculate the structure factor (0.2 mg/ml CSLBs). In the last three lines, the parameters of the Synapsin samples are shown, obtained by fixing b, R_0, and ΔR (indicated by *) and fitting only a. Note that the scattering intensities which were not used in the density calculations differ due to a beam realignment performed during the beamtime.

Sample	a (ph/s/px)	b (ph/s/px)	R_0 (nm)	ΔR (nm)	r^2
1 mg/ml SiO_2 colloids	1718	< 0.01	27.04	1.79	0.9993
0.3 mg/ml CSLBs	556	< 0.01	28.40	2.15	0.9988
0.2 mg/ml CSLBs	804	< 0.01	28.28	2.07	0.9991
6.6 µM Syn + 0.2 mg/ml CSLBs	2136	< 0.01*	28.28*	2.07*	0.9910
9.9 µM Syn + 0.15 mg/ml CSLBs	1189	< 0.01*	28.28*	2.07*	0.9939
13.1 µM Syn + 0.1 mg/ml CSLBs	343	< 0.01*	28.28*	2.07*	0.9977

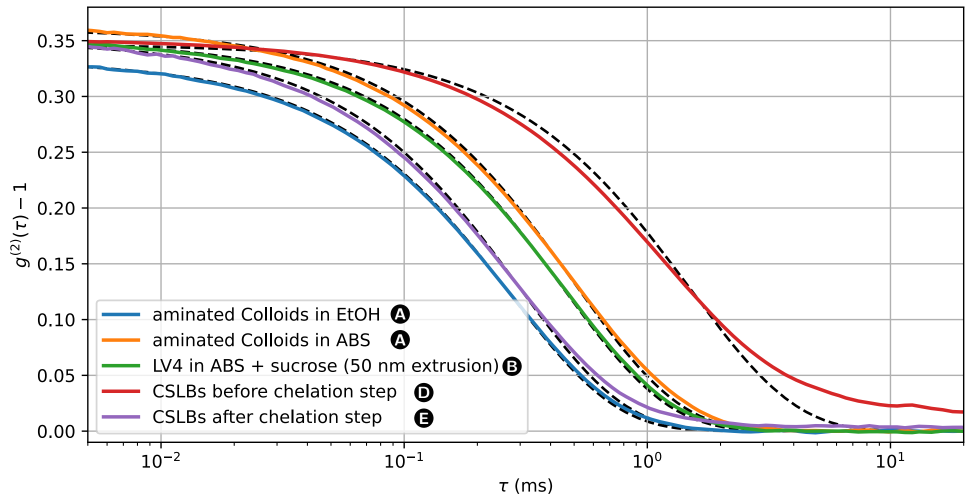

Figure 5: Correlation functions obtained from DLS measurements of CSLBs at various steps of the production process. Dashed black lines indicate the single exponential fit to each correlation function, used to dermine the hydrodynamic radius R_H for each step in the CSLB protocol. The letters indicate the step as detailed in the first figure of the main manuscript.

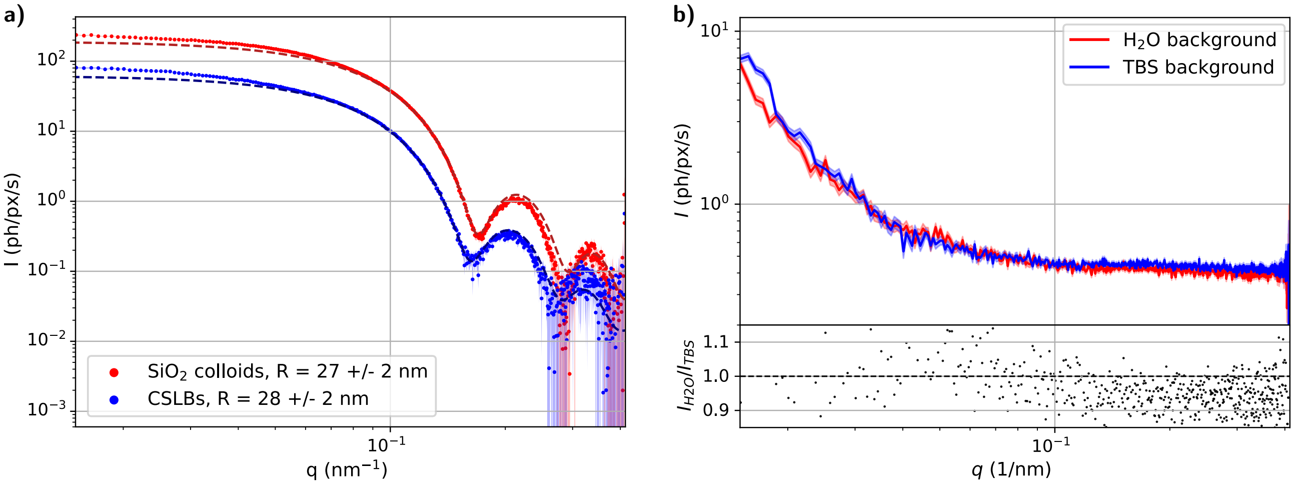

Figure 6: a) Background-subtracted SAXS curves of pure colloids at 1 mg/ml (blue) and containing CSLBs at an unknown concentration (blue) used to determine the CSLB concentration. Background signals (H_2O for colloids, TBS for CSLBs) were adjusted to match the assumed background of the sample measurements and subtracted from the sample curves. A fit with Eq. 4 is also shown (dashed lines), giving $R_{col} = 27(2)$ nm and $R_{CSLB} = 28(2)$ nm, where the uncertainty is determined by the particle polydispersity parameter ΔR. b) Corresponding background curves, used to determine the relative variation of the two background measurements. They remain below approximately 10 %, as the ratio between the two, plotted at the bottom, shows.

FREE DIFFUSION XPCS

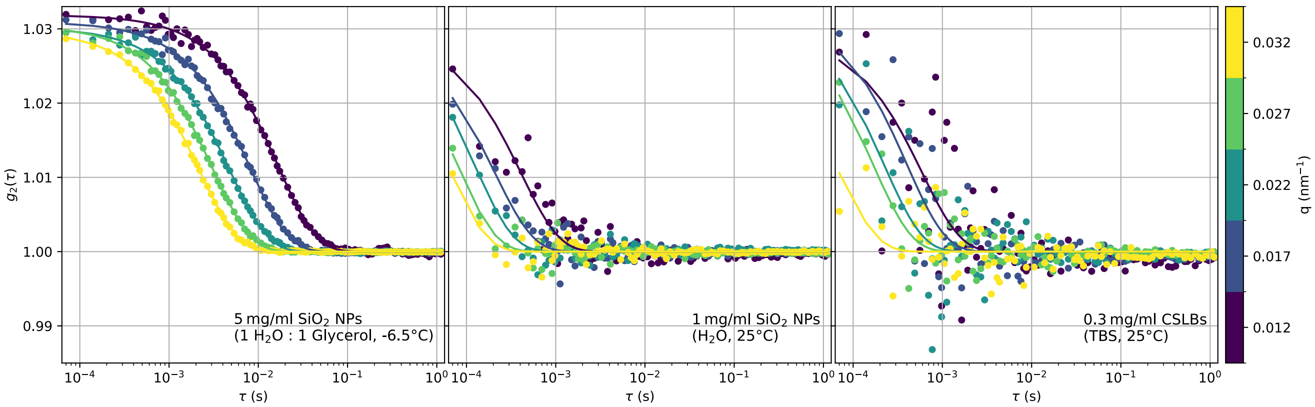

Figure 7: Correlation functions obtained from XPCS measurements of freely diffusing silica colloids and CSLBs. The correlation functions were fitted with a free baseline parameter, whose difference to unity was subtracted for better visualisation. In addition, the speckle contrast β was kept between 0.029 and 0.032, to reduce the degrees of freedom in badly sampled measurements. The range was determined from the fits to the water/glycerol sample.

SYNAPSIN CLUSTER XPCS

The fits to the averaged correlation functions were carried out individually at each measurement position, to account for varying dynamics at different positions along the capillary. All fit parameters of the KWW-fit function were loosely restricted to reasonable values, the speckle contrast was fixed to a range of $\beta \in [0.028, 0.033]$. A least squares fit algorithm was used for all fits. Figures 10, 9, 8 show the correlation functions at representative q-values for all samples at each of the measured positions, excluding all obvious outliers in the measured data (e.g. unrealistic baseline variations).

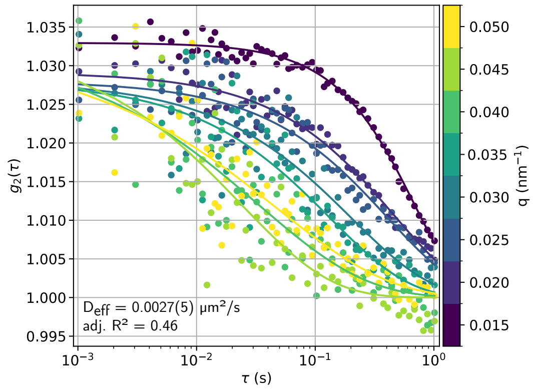

Figure 8: Correlation functions $g^{(2)}(\tau)$ at the reported q-values for the sample containing 6.6 µM Syn and 0.2 mg/ml CSLBs (P/L ratio 1:11). Only a single XPCS train and position was measured at this sample. In addition, we provide the effective diffusion constant D_{eff} obtained from a linear fit (of quality R^2_{adj}) to the fitted relaxation rates (cf. main MS for further details).

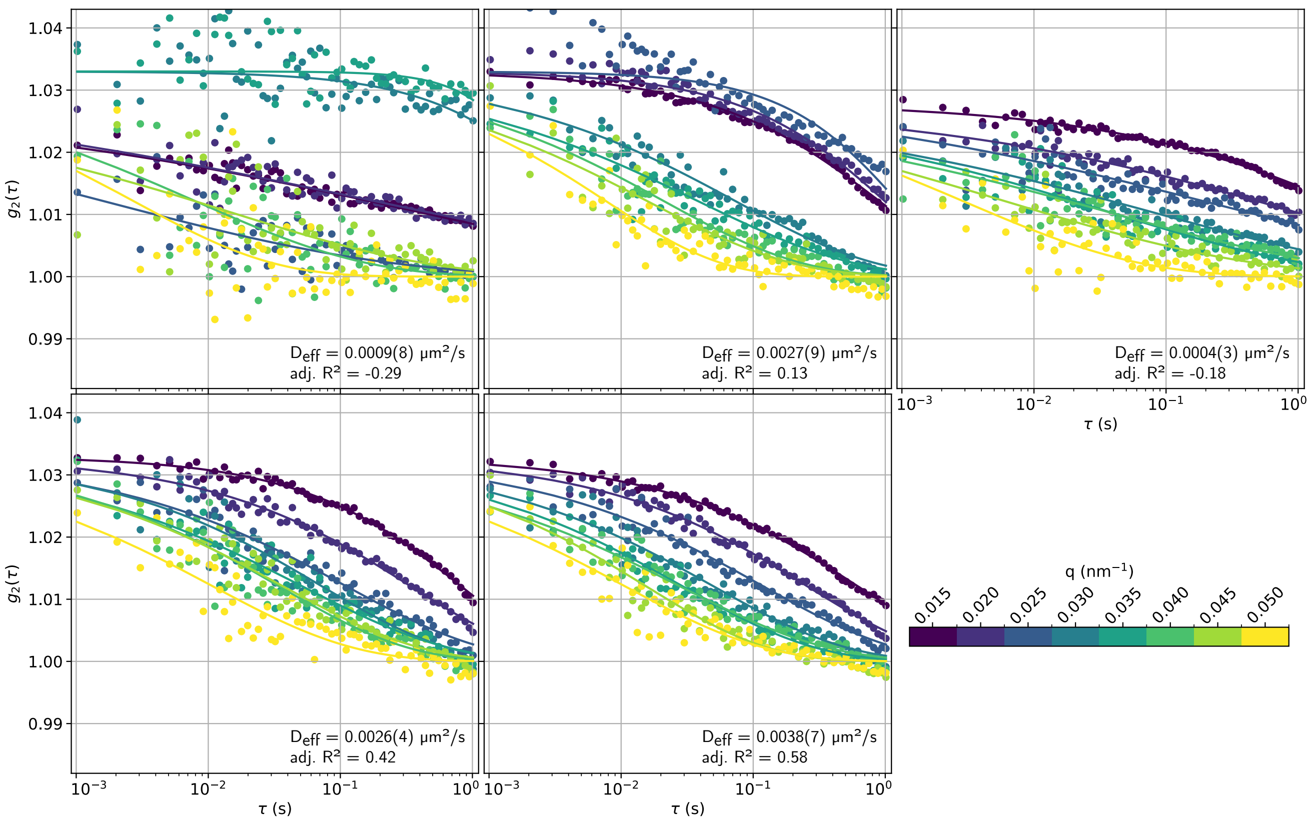

Figure 9: Correlation functions $g^{(2)}(\tau)$ at the reported q-values for the sample containing 9.9 µM Syn and 0.15 mg/ml CSLBs (P/L ratio 1:6). Each correlation function is an average of five individual measurements at each position. In addition, we provide the effective diffusion constant D_{eff} obtained from a linear fit (of quality R^2_{adj}) to the fitted relaxation rates (cf. main MS for further details). For the analysis in the main MS, all values with $R^2_{adj} < 0$ were excluded.

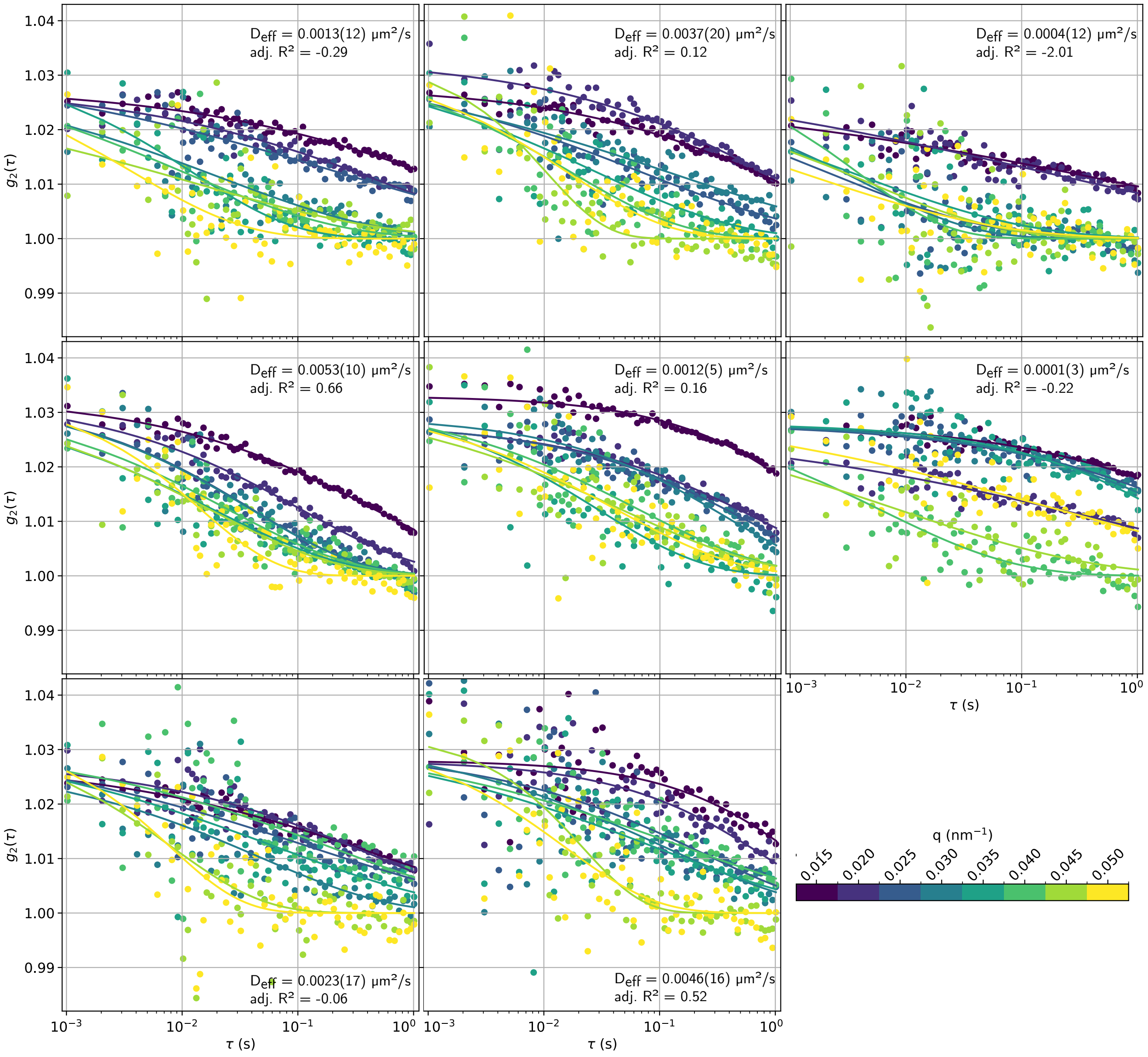

Figure 10: Correlation functions $g^{(2)}(\tau)$ at the reported q-values for the sample containing 13.1 µM Syn and 0.1 mg/ml CSLBs (P/L ratio 1:3). Each correlation function is an average of ten individual measurements at each position. In addition, we provide the effective diffusion constant D_{eff} obtained from a linear fit (of quality R^2_{adj}) to the fitted relaxation rates (cf. main MS for further details). For the analysis in the main MS, all values with $R^2_{adj} < 0$ were excluded.

SYNAPSIN CLUSTER XPCS – SECOND RELAXATION

In two protein-CSLB samples (PL 1:3 and 1:6), an additional, second, relaxation was observed at long timescales, at around $\tau = 10\,\mathrm{s}$. This timescale was not observed in the third sample at P/L 1:11. To quantify the observed dynamics, the analysis from before was repeated on datasets covering timescales from 50 ms to 50 s (1200 frames, approx. 330 kGy total). The values obtained from averages at each position are shown for a single q-value ($q = 0.04\,\mathrm{nm}^{-1}$) in Figure 11a,b. No obvious outliers were identified and correlation functions from all positions were fitted with KWW-stretched exponentials for subsequent parameter analysis. The resulting parameter estimates (weighted arithmetic mean) for Γ and α are plotted against q^2 in Figures 11c and 11d, respectively.

The fitted values of Γ show extremely slow relaxation rates and no significant variations of the relaxation rate across the analysed q-range, with $\Gamma \approx 0.02\,\mathrm{s}^{-1}$. The KWW-exponent α varies between the two samples, but remains flat across the entire sampled q-range and also displays a low variance between different positions. While the motion is superdiffusive with $\alpha \approx 1.2$ at the lower P/L ratio of 1:6, a KWW-exponent closer to 1.0 is observed at the higher P/L ratio (1:3), indicating diffusive behaviour. The largely q-independent scaling of the relaxation rate suggests that the effect is caused by motion of the entire cluster, for example due to convective effects or vibrations inside the capillary, rather than by individual CSLBs. Furthermore, similarly superdiffusive motion was previously found to occur in a tumbling motion of a cluster of tracer particles (5). Cluster motion also explains the variety of different correlation functions in Figure 11c, as each measurement spot probes a different position, where the local dynamics likely varies depending on the local cluster structure and thermodynamic conditions. The slight difference of KWW-exponent between the two samples, however, also suggests that the cluster motion is influenced by the composition of the cluster.

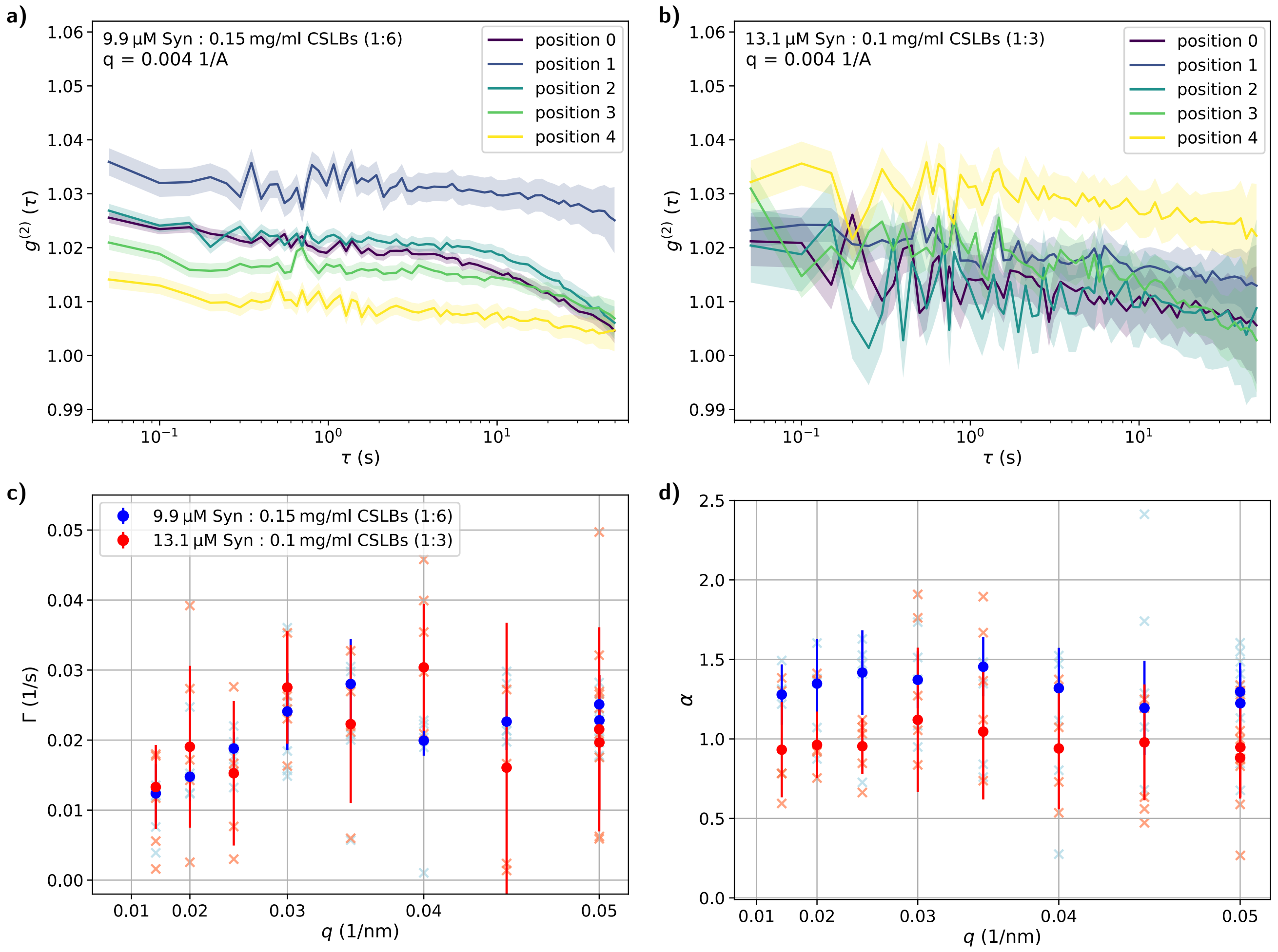

Figure 11: (a, b) Cyclic averages of the correlation function for the sample at P/L ratio 1:6 (a) and P/L ratio 1:3 (b) at every measurement position. The dynamics at different positions appear dissimilar and are thus fitted individually without taking a prior average. Relaxation rates, however, are similar with differences arising due to varying contrast levels, as the subsequent analysis shows. c) Relaxation rates for the second, slow relaxation in protein samples at P/L ratios 1:3 (red) and 1:6 (blue), plotted against q (axis scales with q^2). KWW exponential decays were fitted to the averaged correlation functions for $\tau > 1$ s for individual measurement positions (pale crosses, error bars not shown). Individual measurements were subsequently combined using a weighted arithmetic mean (bright circles). A slight increase in the relaxation rate is observed at low q, but the relaxation rate remains almost constant for the most part of the covered q-range. d) Corresponding KWW-exponents α plotted against q. They reveal diffusive motion ($\alpha \approx 1$) at a 1:3 P/L ratio and slightly superdiffusive motion ($\alpha \approx 1.4$) at a 1:6 P/L ratio.

REFERENCES

1. Jankowski, M., et al., 2023. The complex systems and biomedical sciences group at the ESRF: Current status and new opportunities after extremely brilliant source upgrade. *Nucl. Instrum. Methods. Phys. Res. B* 538:164. https://doi.org/10.1016/j.nimb.2023.02.034.

2. Raimondi, P., et al., 2023. The Extremely Brilliant Source storage ring of the European Synchrotron Radiation Facility. *Commun. Phys.* 6. https://doi.org/10.1038/s42005-023-01195-z.

3. Zinn, T., et al., 2018. Ultra-small-angle X-ray photon correlation spectroscopy using the Eiger detector. *J. Synchrotron Radiat.* 25:1753. https://doi.org/10.1107/S1600577518013899.

4. Chushkin, Y., et al., 2022. Probing Cage Relaxation in Concentrated Protein Solutions by X-Ray Photon Correlation Spectroscopy. *Phys. Rev. Lett.* 129:238001. https://doi.org/10.1103/PhysRevLett.129.238001.

5. Czajka, T., et al., 2023. Lipid vesicle pools studied by passive X-ray microrheology. *Eur. Phys. J. E* 46:123. https://doi.org/10.1140/epje/s10189-023-00375-7.

6. Nicolas, J.-D., S. Aeffner, and T. Salditt, 2019. Radiation damage studies in cardiac muscle cells and tissue using microfocused X-ray beams: experiment and simulation. *J. Synchrotron Rad.* 26:980. https://doi.org/10.1107/S1600577519006817.

7. Hubbell, J., and S. Seltzer, 1995. Tables of X-Ray Mass Attenuation Coefficients and Mass Energy-Absorption Coefficients, NIST Standard Reference Database 126. http://www.nist.gov/pml/data/xraycoef/index.cfm.

8. Reiser, M., et al., 2022. Resolving molecular diffusion and aggregation of antibody proteins with megahertz X-ray free-electron laser pulses. *Nat. Commun.* 13:5528. https://doi.org/10.1038/s41467-022-33154-7.

9. Als-Nielsen, J., and D. McMorrow, 2011. Elements of modern X-ray physics. John Wiley & Sons, West Sussex, UK, 2 edition.
